# Byways of Medical Relief

**Published:** 1912-03-02

**Authors:** 


### BYWAYS OF MEDICAL RELIEF.*

(Contributions are invited to this column.)

### VIII.—Little Homes for the Aged.

The result of efforts undertaken on behalf of the aged is often disappointing to the benevolent. There is a great demand for such homes. Generally the managers of them make a charge of at least ten shillings a week, a sum which is insufficient to meet the expenses of rent, food, attendance and other necessaries. But the charitable who support such homes, and the superintendents chosen to administer them often fail to appreciate to the full the difficulties of the enterprise. They are a failure from the highest point of view, that of the contentment of the inmates, because the problems they present are unintelligently encountered. The approach of age represents a pathological, mental and moral crisis in the life of the individual. Some old people retain their faculties and their happy temper of mind to the end. Others become morose and even morbidly objectionable, after a useful life. May we not observe the actual manufacture of the latter types in the course of inspecting a workhouse infirm ward, or an ill-managed home?

### Vital Importance of Light Diet.

Many of the ailments which afflict persons of sedentary habits who are past the prime of life are digestive. Hence the problem of supplying inmates of such homes with food which they can readily assimilate is the most pressing of all. The perpetual craving for food which often afflicts old people is partly a habit, partly a symptom of stomach uneasiness. The meals should be light and very leisurely. The less in actual bulk they can be taught to consume as life goes on, the better, but restriction of appetite must be a purely voluntary act, and formal allowancing is out of the question. It is better to spread the principal meal into three light courses, with longish intervals between, than to let the appetite be sated all at once. Under this precaution the wolf hunger which is a great torment to some weak digestions dies naturally away, and the meal is enjoyed without a surfeit. Prevalence of indigestion and constipation, with their natural consequence—recourse to purgatives, should be considered an indication of failure in the diet.

Fruit is an essential part of the diet of old and young, and is generally much liked. There is often a great craving for sugar, and some form of sweetmeat ought to be a regular form of indulgence. Hard meat, heavy puddings, pastry, salt fish, etc., must be excluded from the bill of fare, but on the other hand old people appreciate tasty sauces and need not be denied the sharp flavourings for which their jaded sense of taste gives them a penchant. The use of digestive tea and the special coffees which can now be obtained free from over stimulating properties would relieve many a sufferer from uneasy symptoms. The tannin in ordinary tea acts often injuriously on the mucous membrane in the aged and disturbs the whole alimentary tract.

To keep the inmates of these little homes well nourished and yet free from the burden of ill-digested food is the first step towards keeping them content. The next step is to encourage them to take regular exercise almost irrespective of weather. To get all the inmates into the air twice a day may seem to some managers a gigantic and unnecessary task. And yet if the habit can be once established it will relieve more than any other the boredom of existence, and prove a safeguard against many unsafe mental conditions. Those who cannot walk should be taken out in a chair, and in really bad weather some substitute for walking exercise should be supplied, either in simple movements of the limbs, or better still in games which demand a little moving about. When it is remembered how large a part is played by games in whiling away the tedium of idle hours for the rich it may be seen how readily the poor would respond to the stimulus of such recreations. Without work and without play man is indeed a dull dog.

Some form of employment should be secured to the most comatose members of this little society. And to this end the inmates should be provided each with their own little table and if possible their own light. In building new quarters for this purpose thought should be taken of the orderly habits of old people and rooms should be planned so that all can make sure of a comfortable seat, reserved for their exclusive use, and of a private locker, fitted with key, for the aged are ever suspicious.

### The Personal Element.

Lastly, when all is done, the aged cannot prosper without honour. It is not enough to remove bodily discomforts, there must be an appeal to the mind and the heart. It is the little signs of consideration, the patient redress of some tiny grievance, it is these which turn "inmates" to friends, and set the blue bird singing in hearts which have nothing further to cheer their imaginations.

For those who can find the key to the hearts of the aged there is much joy in store. When the toils of life are over the veterans have often a singular and disinterested gift of sympathy to spare for those who are still in the forefront of the battle. They have all the tenderness and sweet frankness of little children, with an understanding mind which the child can never possess. Their love is one of the most fragrant gifts of the spirit. To serve them is to be brought very close to the divine secrets.

The Aged Pilgrims' Friend Society has four homes for the aged; for Roman Catholics there are homes under the Little Sisters of the Poor, and at Nazareth House, Hammersmith; for soldiers and sailors there is Princess Christian's Home at Portsmouth; and we may also mention St. Cyprian's Home for Aged Poor Women at Little Park Street, N.W., and St. Peter's Harbour, 10 Greville Place, N.W.

* Previous articles of this series have appeared in The Hospital of October 7 and 21, November 18, December 2, January 6, February 10 and 17.

